# The relationship between blood metabolites of the tryptophan pathway and kidney function: a bidirectional Mendelian randomization analysis

**DOI:** 10.1038/s41598-020-69559-x

**Published:** 2020-07-29

**Authors:** Yurong Cheng, Yong Li, Paula Benkowitz, Claudia Lamina, Anna Köttgen, Peggy Sekula

**Affiliations:** 1 Department of Biometry, Epidemiology and Medical Bioinformatics, Institute of Genetic Epidemiology, Faculty of Medicine and Medical Center - University of Freiburg, Hugstetter Str. 49, 79106 Freiburg, Germany; 2 Faculty of Biology, University of Freiburg, Freiburg, Germany; 3 Department of Genetics and Pharmacology, Institute of Genetic Epidemiology, Medical University of Innsbruck, Innsbruck, Austria

**Keywords:** Chronic kidney disease, Epidemiology, Risk factors

## Abstract

Blood metabolites of the tryptophan pathway were found to be associated with kidney function and disease in observational studies. In order to evaluate causal relationship and direction, we designed a study using a bidirectional Mendelian randomization approach. The analyses were based on published summary statistics with study sizes ranging from 1,960 to 133,413. After correction for multiple testing, results provided no evidence of an effect of metabolites of the tryptophan pathway on estimated glomerular filtration rate (eGFR). Conversely, lower eGFR was related to higher levels of four metabolites: C-glycosyltryptophan (effect estimate = − 0.16, 95% confidence interval [CI] (− 0.22; − 0.1); *p* = 9.2e−08), kynurenine (effect estimate = − 0.18, 95% CI (− 0.25; − 0.11); *p* = 1.1e−06), 3-indoxyl sulfate (effect estimate = − 0.25, 95% CI (− 0.4; − 0.11); *p* = 6.3e−04) and indole-3-lactate (effect estimate = − 0.26, 95% CI (− 0.38; − 0.13); *p* = 5.4e−05). Our study supports that lower eGFR causes higher blood metabolite levels of the tryptophan pathway including kynurenine, C-glycosyltryptophan, 3-indoxyl sulfate, and indole-3-lactate. These findings aid the notion that metabolites of the tryptophan pathway are a consequence rather than a cause of reduced eGFR. Further research is needed to specifically examine relationships with respect to chronic kidney disease (CKD) progression among patients with existing CKD.

## Introduction

The kidneys play a major role in maintaining homeostasis in the human body by regulating the excretion of both endogenous and exogenous molecules below a certain mass, such as many metabolites. The estimated glomerular filtration rate (eGFR) is the most widely used measure of kidney function^1^. Chronic kidney disease (CKD) is defined as a persistent reduction of eGFR or kidney damage and is associated with a higher risk of comorbidities and mortality^2^. With an increasing prevalence of currently 10–15% worldwide, it represents a global health burden^2,3^. Therapeutic options are limited and aim to stop CKD progression, thereby attempting to reduce risk of end-stage kidney disease (ESKD), early death and comorbidities^2^.

Blood and urine metabolite levels are altered in the presence of CKD, making evaluations of the metabolome of interest in nephrology^4–6^: while some metabolites may represent filtration markers such as creatinine and thus may allow for improving the estimation of GFR, other metabolites may provide complementary insights into renal physiology by acting as a readout of active tubular secretion or reabsorption. Furthermore, metabolites might be causally related to development and progression of CKD, such as glucose in diabetic nephropathy. A central question in nephrology therefore is whether alterations in metabolite levels represent a cause or a consequence of CKD.

**Figure 1:**
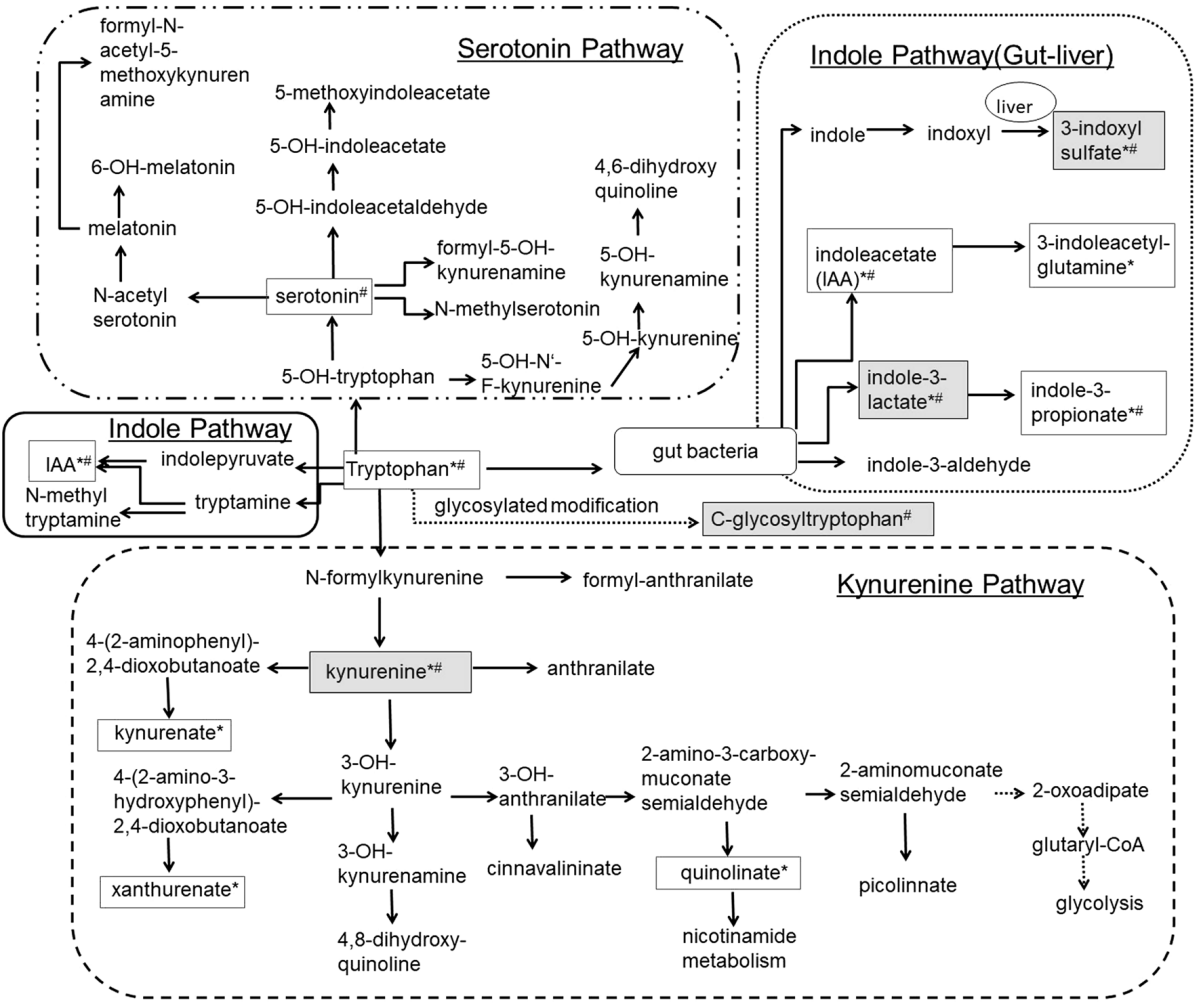
Metabolites and sub-pathways of the tryptophan pathway. For Metabolites with a frame necessary data were available and evaluated in this MR study to evaluate the impact of metabolites on eGFR (*) and/or the impact of eGFR on metabolites (#). Furthermore, frames with grey background mark metabolites that show significant results in our analysis.

Recent advances in mass methodology^7–9^ allow for comprehensive studies of the metabolome and its relation to kidney function^4,5^. A common theme from the reported literature is the association between blood levels of metabolites of the tryptophan pathway (i.e. tryptophan and its down-stream products) and kidney function and disease (Supplementary Table 1). Tryptophan is an essential amino acid in humans^10,11^. While less than 1% of tryptophan is actually used for protein biosynthesis, the major proportion serves as a biosynthetic precursor of microbial and host metabolites along different pathways, including the serotonin, the kynurenine, and the indole pathways (Fig. 1)^10–13^.

We previously reported that several ratios of serum metabolite levels containing tryptophan that had been quantified by a targeted measurement technique in a population-based study were significantly associated with eGFR^14^. In a follow-up project, the serum kynurenine-to-tryptophan ratio was significantly associated with annual change in eGFR and incident CKD^15^. This specific ratio presumably reflects the enzymatic activity of indoleamine 2,3-dioxygenase and tryptophan 2,3-dioxygenase, responsible for the conversion of tryptophan to kynurenine via N-formylkynurenine. Furthermore, we evaluated nine metabolites of the tryptophan pathway based on non-targeted measurements in a similar study, and identified C-glycosyltryptophan (also known as C-mannosyltryptophan) to be associated with eGFR and CKD, as well as with prospective endpoints of eGFR decline, incident CKD and ESKD^16^.

Overall, studies evaluating associations between metabolites of the tryptophan pathway and kidney function or disease provide a mixture of significant and non-significant associations. Potential reasons relate to variability in measurement techniques (e.g. liquid chromatography, mass spectrometry) and coverage of measurements (e.g. targeted vs non-targeted, number of metabolites measured) as well as to variability in analysis methods (e.g. comparison of levels between groups, analysis of association with eGFR or incident CKD). Moreover, these findings originate only from observational studies of different designs, sizes and characteristics. Observational associations do not allow for establishing causal relationships between a metabolite and kidney function or disease, and can be limited in their ability to distinguish between cause and consequence^6,17^.

Experimental studies also support a relationship between metabolites of the tryptophan pathway and kidney function: For example, an excess in the conversion of tryptophan to N-formylkynurenine was shown to promote renal ischemia–reperfusion injury in mice^18^. Korstanje et al*.* showed that the knockdown of kynurenine 3-mono-oxygenase gene expression in zebrafish and systemic deletion of the gene in mice led to a proteinuric phenotype^19^. C-mannosylation as a specific type of glycosylation was shown to be increased under hyperglycemic conditions, prompting a hypothesis of its pathological role in the development of diabetic complications^20^. Another example, serum 2-(α-mannopyranosyl)-l-tryptophan was found to be an accurate measure of renal function in rats^21^. Kobayashi et al. finally demonstrated in an untargeted metabolite screen of rats with and without CKD that metabolites of the tryptophan pathway might be used to detect early stages of CKD^22^. Thus, complementary lines of evidence connect metabolites of the tryptophan pathway to kidney function and disease.

Here, we designed a study to address the following question: Are tryptophan, kynurenine or other metabolites of the tryptophan pathway in blood causally related to kidney function, or does reduced kidney function influence blood metabolite levels? We used a bidirectional Mendelian randomization (MR) approach allowing inference of causality^17,23^. An extension of this approach conveniently allows for the usage of publicly available summary statistics from published genetic association studies^24^.

## Results

More than 40 metabolites qualified as down-stream products of tryptophan in humans (Fig. 1, Supplementary Information 1). The search for relevant published studies focused on genome-wide association studies (GWAS) of these metabolites. In addition, we searched for GWAS of kidney function. Five published studies including four GWAS of metabolites and one large GWAS of eGFR were identified and used as the basis of this MR analysis (“Methods”, Supplementary Table 2).

Based on published results of selected GWAS, genetic variants were identified as instruments for the respective trait (see “Methods” for details). The number of selected instruments per trait ranged from one instrument to 22 instruments for metabolites and resulted in 73 instruments for eGFR (Supplementary Table 3). Selected instruments for a given trait are not only independent of each other but with few exceptions also across traits (*R*^2^ < 0.2, Supplementary Table 3). Whenever possible, the strength of genetic variants was estimated in terms of *F* statistic and ranged between 95 and 631 for eGFR and between 18 and 135 for metabolites (Supplementary Table 3)^25,26^.

### Mendelian randomization analysis reveals four significant effects

Overall, 18 MR analyses could be conducted (Supplementary Table 3): For the direction: metabolite → kidney function, ten different metabolites of the tryptophan pathway and the kidney function measure eGFR were evaluated. Data from more than one GWAS were available for kynurenine (N = 2) and indole-3-propionate (N = 3). For the other direction: kidney function → metabolite, MR analysis was carried out for eGFR and eight metabolites from one GWAS.

Reported pairwise correlations of metabolites ranged from − 0.24 for tryptophan and C-glycosyltryptophan to 0.72 for kynurenate and xanthurenate, with many of them close to zero (Supplementary Fig. 1).

In the MR analysis with metabolites as exposure and eGFR as the outcome, none of the reported effect estimates reached significance after correction for multiple testing (*p* > 2.78e−03; Table 1). The effect of xanthurenate on eGFR was nominally significant (effect estimate = − 0.0086; *p* = 2.9e−02). Conversely, eGFR affected the levels of four metabolites: C-glycosyltryptophan (effect estimate = − 0.16, 95% confidence interval [CI] (− 0.22; − 0.1); *p* = 9.2e−08), kynurenine (effect estimate = − 0.18, 95% CI (− 0.25; − 0.11); *p* = 1.1e−06), 3-indoxyl sulfate (effect estimate = − 0.25, 95% CI (− 0.4; − 0.11); *p* = 6.3e−04) and indole-3-lactate (effect estimate = − 0.26, 95% CI (− 0.38; − 0.13); *p* = 5.4e−05; Table 1). The negative estimates for all four metabolites indicate that higher levels of metabolites are a consequence of lower eGFR. Further interpretation of effect estimates with regards to their magnitude was not possible due to the nature of metabolite measurements (semi-quantitative, unitless) and different handling of measurements (transformation) in the underlying studies (Supplementary Table 2).

**Table 1:** Main results of Mendelian randomization analyses.

Exposure: metabolite, unitless		Outcome: eGFR, mL/min/1.73 m^2^ (Pattaro et al*.* 2016^90^)						
		N SNPs	Main analysis results^a^			Main sensitivity analysis results		
			Effect Estimate (SE)	95% CI	*p*	Egger regression		MR-PRESSO global test *p*
						Effect Estimate (SE)	Intercept (SE)	
**Direction: metabolite → kidney function**								
3-indoxyl sulfate	Rhee et al*.* 2013^86^	1	− 0.0012 (0.0041)	(− 0.0092; 0.0068)	7.68E−01	NA	NA	NA
indoleacetate	Shin et al. 2014^87^	2	0.00001 (0.0375)	(− 0.0735; 0.0735)	1.00E + 00	NA	NA	NA
indoleacetylglutamine	Long et al. 2017^89^	1	0.0004 (0.0058)	(− 0.0110; 0.0117)	9.51E−01	NA	NA	NA
indole-3-lactate	Shin et al*.* 2014^87^	1	− 0.08 (0.05)	(− 0.18; 0.01)	8.11E−02	NA	NA	NA
indole-3-propionate	Rhee et al. 2013^86^	1	− 0.0029 (0.0044)	(− 0.0116; 0.0058)	5.13E−01	NA	NA	NA
indole-3-propionate	Shin et al*.* 2014^87^	2	0.01 (0.02)	(− 0.03; 0.06)	5.14E−01	NA	NA	NA
indole-3-propionate	Long et al*.* 2017^89^	1	− 0.01 (0.01)	(− 0.03; 0)	5.73E−02	NA	NA	NA
kynurenate	Long et al. 2017^89^	1	0.01 (0.01)	(0; 0.02)	7.74E−02	NA	NA	NA
kynurenine	Rhee et al. 2013^86^	1	<|0.00001| (0.0034)^b^	(− 0.0067; 0.0067)	1.00E + 00	NA	NA	NA
kynurenine	Shin et al*.* 2014^87^	5	− 0.04 (0.04)	(− 0.12; 0.05)	3.69E−01	0.03 (0.16)	− 0.0012 (0.0027)	2.94E−02
tryptophan	Shin et al*.* 2014^87^	22	0.04 (0.04)	(− 0.03; 0.11)	3.04E−01	− 0.23 (0.22)	0.0015 (0.0012)	8.61E−01
quinolinate	Rhee et al*.* 2013^86^	2	0.0026 (0.0032)	(− 0.0038; 0.0089)	4.29E−01	NA	NA	NA
xanthurenate	Rhee et al. 2013^86^	1	− 0.0086 (0.0039)	(− 0.0162; − 0.0009)	2.92E−02	NA	NA	NA

Outcome: metabolite, unitless		Exposure: eGFR, mL/min/1.73 m^2^ (Pattaro et al. 2016^90^)						
		N SNPs	Main analysis results^a^			Main sensitivity analysis results		
			Effect Estimate (SE)	95% CI	*p*	Egger regression		MR-PRESSO global test *p*
						Effect Estimate (SE)	Intercept (SE)	
**Direction: kidney function → metabolite**								
3-indoxyl sulfate	Shin et al*.* 2014^87^	73	− **0.25 (0.07)**	**(**− **0.4**; − **0.11)**	**6.26E**−**04**	− 0.05 (0.27)	− 0.0016 (0.0021)	2.46E−01
C-glycosyltryptophan	Shin et al*.* 2014^87^	73	− **0.16 (0.03)**	**(**− **0.22**; − **0.1)**	**9.22E**−**08**	− 0.14 (0.12)	− 0.0002 (0.0009)	1.49E−01
indoleacetate	Shin et al*.* 2014^87^	73	− 0.05 (0.07)	(− 0.19; 0.09)	5.03E−01	− 0.19 (0.26)	0.0011 (0.0020)	1.20E−02
indole-3-lactate	Shin et al. 2014^87^	73	− **0.26 (0.06)**	**(**− **0.38**; − **0.13)**	**5.43E**−**05**	− 0.35 (0.24)	0.0007 (0.0018)	**3.00E**−**04**
indole-3-propionate	Shin et al*.* 2014^87^	73	− 0.05 (0.08)	(− 0.2; 0.1)	5.26E−01	− 0.18 (0.29)	0.0010 (0.0022)	1.02E−01
kynurenine	Shin et al*.* 2014^87^	73	− **0.18 (0.04)**	**(**− **0.25**; − **0.11)**	**1.07E**−**06**	− 0.18 (0.13)	0.0001 (0.0010)	3.17E−01
serotonin	Shin et al. 2014^87^	73	− 0.13 (0.08)	(− 0.28; 0.02)	7.85E−02	0.03 (0.28)	− 0.0013 (0.0021)	2.27E−02
tryptophan	Shin et al*.* 2014^87^	73	0.04 (0.02)	(− 0.01; 0.09)	1.08E−01	0.02 (0.09)	0.0002 (0.0006)	3.20E−02

Note of caution: Because of unitless metabolite levels and differences in applied transformations of traits in underlying genome-wide association studies, effect estimates and confidence intervals are limited in their interpretation and comparison.

*SE* standard error, *CI* confidence interval. Association results marked in bold passed significance threshold corrected for multiple testing: 0.05/(10 + 8) = 2.78E−03 (A: 10 Metabolites B: 8 Metabolites).

^a^The Wald ratio estimator was used for effect estimation if one single instrument was available, and the inverse variance weighted estimation method if more than one instrument was available.

^b^Due to numerical constraints, no more exact estimate can be provided; estimated effect is within the range of − 0.00001 and + 0.0001. NA: Respective analysis was not feasible due to small number of instruments (< 3).

### Sensitivity analyses support a role of low eGFR in elevated blood metabolites of the tryptophan pathway

As outlined in the “Methods” section, additional analyses were performed to evaluate the robustness of the results and to ensure the absence of any major violations of the assumptions underlying MR (Supplementary Table 4, Supplementary Fig. 2). Overall, the results were consistent. Focussing on significant observations:

For C-glycosyltryptophan and kynurenine, all estimates of the effects using additional estimation methods were direction-consistent showing little variation (range of effect estimates: − 0.14; − 0.21, Supplementary Table 4, Supplementary Fig. 3). Neither the estimate of Egger intercept nor other statistics indicated any violations of the MR assumptions. Similarly, there was also no indications for any violations for 3-indoxyl sulfate, although the range of effect estimates was wider (− 0.05; − 0.29).

In contrast, the heterogeneity test (*p* = 4.3E−04) as well as the MR-PRESSO test (*p* = 3.0E−04) indicated some violations of underlying assumptions for indole-3-lactate (Supplementary Table 4). Since MR-PRESSO implicated two SNPs (marked in Supplementary Fig. 3-D) as outliers, an additional analysis was conducted after exclusion of these two SNPs. The outlier-corrected effect estimate of eGFR on indole-3-lactate, however, differed only marginally from the estimate of the main analysis (effect estimates: − 0.24 vs − 0.26, respectively) as well as results of other methods (range of effect estimates: − 0.23; − 0.48) and therefore supported the relationship despite the indications for a violation of the MR assumption that instruments are only associated with the outcome through the exposure.

In addition to sensitivity analyses using statistical methods, we assessed the validity of the results from a biological perspective. Besides the potential issues caused by the inclusion of pleiotropic SNPs, we also addressed the potential issue of the inclusion of weak instruments or instruments with spurious associations by utilizing differently defined subsets of SNPs (“Methods”, Supplementary Table 5). The results of these additional sensitivity analyses were quite similar in comparison to the main analysis (Supplementary Table 4), supporting the validity of the main results on the relationship of eGFR with these four blood metabolites.

## Discussion

Here, we carried out a bidirectional two-sample MR analysis to study whether blood metabolite levels of the tryptophan pathway causally affect the kidney function measure eGFR, or whether kidney function affects levels of these metabolites in blood. After correction for multiple testing, the principal finding of this study indicates that lower eGFR causes higher levels of the four metabolites kynurenine, C-glycosyltryptophan, 3-indoxyl sulfate and indole-3-lactate in blood, whereas the opposite analysis did not support a causal role of these metabolites on eGFR.

Kynurenine is synthesized from tryptophan via N-formylkynurenine^10,13,27^. Levels of kynurenine are elevated in ESKD patients and a toxic effect has been discussed^28,29^. Several cross-sectional and case–control studies reported increased levels of kynurenine in patients with reduced kidney function thereby supporting our finding^16,30–35^.

In contrast to kynurenine, levels of tryptophan are depleted in patients with reduced kidney function^33,36–38^. This might indicate an increased activity of enzymes converting tryptophan to kynurenine via N-formylkynurenine^18,27,37–44^. Indeed, observational studies in humans reported an association between the tryptophan/kynurenine ratio and lower eGFR^15,31,38^. Our results do not indicate a causal relationship between tryptophan and kidney function of any direction. As there are no genetic association results for metabolite ratios such as the tryptophan/kynurenine ratio available, no MR analysis could be conducted with respect to this reported observation.

C-glycosyltryptophan (or C-mannosyltryptophan) results from a post-translational modification of tryptophan by linking a sugar via a carbon–carbon bond^45,46^. Certain post-translational modifications such as carbamylation have been linked to different chronic conditions including renal failure^47^. Studies in humans showed consistently increased levels of C-glycosyltryptophan in people with decreased eGFR^16,48–52^. Moreover, studies of humans and animal models suggested C-glycosyltryptophan to be a good indicator of renal function with more favourable properties compared to serum creatinine^16,21,51,53^. Thus, our finding that eGFR has an impact on blood levels of C-glycosyltryptophan is supported by published results. Although there is no literature to support the opposite direction in general, some prospective studies in humans (population-based or diabetic cohorts) showed that higher levels of C-glycosyltryptophan are associated with an increased risk for eGFR decline, even after adjustment for baseline eGFR^16,50,54^. In addition, Sekula et al*.* reported that the significant risk for ESKD in CKD patients was attenuated after adjustment for baseline eGFR and lost significance after adjustment for baseline measured GFR^16^. While our study did not indicate a causal relationship of C-glycosyltryptophan on eGFR in general, higher levels of C-glycosyltryptophan may still be of relevance in patients with advanced CKD.

The other two metabolites, 3-indoxyl sulfate and indole-3-lactate, belong to the indole pathway and are synthesized by gut bacteria^11,55,56^. Disturbances of the microbiome have been associated with many chronic diseases such as CKD^11,57–66^, and a bidirectional relationship between host and microbiome has been reported^56,60,67,68^.

3-Indoxyl sulfate, also known as indican, is a known uremic toxin with highly elevated levels in ESKD patients^28,29,44,69–71^. Its toxic effect on kidney function as well as other organs and pathways is still being discussed^6,72^. Several observational studies on humans with and/or without CKD reported associations between higher levels of 3-indoxyl sulfate and lower eGFR^16,30,32,33,35,73–76^. Increased levels of 3-indoxyl sulfate were also observed in rats with autosomal dominant polycystic kidney disease (ADPKD) versus control rats^77^. All considered, the consistent results of these studies support our finding on the impact of eGFR on 3-indoxyl sulfate but not on its discussed toxic effect on kidney function.

In contrast, there is not much data available for indole-3-lactate. In a previous association study, higher levels of indole-3-lactate were found to be cross-sectionally associated with lower eGFR^16^. In an elderly population, indole-3-lactate was found to be associated not only with muscle composition but also with the renal function markers serum creatinine and blood urea nitrogen^78^. Quite recently, a study group reported that increased levels of indole-3-lactate were found to be associated with lower eGFR in a cohort of patients with ADPKD^79^. Altogether, study results support a causal relationship of low eGFR on blood indole-3-lactate levels.

Strengths of this MR study are the availability of multiple metabolites within a specific pathway, the use of various publicly available datasets based on rigorously conducted GWAS meta-analyses, the conduct of statistically as well as biologically motivated sensitivity analyses, and consistent results pointing into one direction. The analysis of summary statistics also has some disadvantages and depends upon the quality and power of included studies. All studies incorporated in this MR study are observational and vary with respect to several characteristics:

Besides differences in general study characteristics and in analysis strategies (e.g. transformation of traits), groups used different laboratory platforms (genetics, metabolomics) with measurements of varying quality (e.g. semi-quantitative measurements), limiting their comparability. It also prevented pooling of studies to gain additional power. Furthermore, interpretation of effect estimates is thus hampered as well as their comparison to reported associations. Here, interpretation of results is restricted to the direction of effects.

Furthermore, the coverage of evaluated metabolites varied over time because of advances in metabolite quantification. In combination with only partial reporting and sharing of analysis results, the search for relevant GWAS for studies such as ours is limited in itself. In consequence, the MR analyses were restricted to a subset of metabolites of the tryptophan pathway. Still, analyses could be conducted for several metabolites and data from more than one source was partially available to evaluate relationship for some metabolite-kidney function combinations.

The population sizes of selected studies were quite different, with the sample size for eGFR being much larger than the currently largest reported studies for the metabolites (Supplementary Table 2), which may raise concern about differential statistical power for bidirectional MR. However, the tight link between the genome and the metabolome does not require a similar study size as needed for the evaluation of a complex trait such as eGFR, as evidenced in large *F* statistics of the selected metabolite instruments. Since levels of metabolites are often influenced by a single gene, the low number of instruments for metabolites is therefore not unexpected. In contrast, we used several SNPs as instruments for eGFR reflecting its complex nature.

A limitation inherent to all MR-studies is that effect estimates might be biased in the presence of weak instruments that do not reflect lifelong exposures to altered levels of the factor of interest such as eGFR or a metabolite strongly enough. To ensure inclusion of strong instruments, we addressed this challenge by selecting only genome-wide significant genetic variants^80^. In addition, we evaluated the strength of instruments by estimating the *F* statistics whenever possible. Instruments of eGFR were stronger than those of metabolites because of the large sample size of the eGFR GWAS, but metabolite instruments still had large *F* statistics. Sensitivity analyses restricting instruments to SNPs with the very strongest or – in case of eGFR – strong and replicated associations supported the main findings based on all identified instruments.

Another concern in MR-studies is the presence of pleiotropy, such that genetic instruments are associated with the studied outcome directly or via other paths. Here, we also observed that there were few instances in which the same or a closely linked genetic variant was selected as instrument for more than one metabolic trait. For metabolites, this is possible even in the absence of pleiotropy, because, for instance, a genetic variant in an enzyme can be associated both with its metabolic substrate and its product. We addressed this potential limitation by using a variety of additional, more robust estimation methods^81^ as well as by using subsets of SNPs thereby reducing risk of pleiotropy. However, sensitivity analyses could be conducted only if a sufficient number of instruments for the respective exposure was available. Although we cannot completely rule out any pleiotropy, the results of our sensitivity analyses supported the main finding that eGFR is related to blood levels of tryptophan metabolites.

Lastly, our findings are based on summary statistics from population-based cohorts with relatively few individuals with advanced CKD. While our results do not indicate that metabolite levels of the tryptophan pathway are causally related to eGFR, our data are not sufficient to address the question whether these levels are related to CKD progression in patients with pre-existing CKD. Moreover, limited data did not allow to address other traits of kidney function (e.g. urinary albumin-to-creatinine ratio, UACR).

In summary, our study supports that lower eGFR causes higher blood levels of four metabolites of the tryptophan pathway kynurenine, C-glycosyltryptophan, 3-indoxyl sulfate, and indole-3-lactate, whereas higher levels of these metabolites did not cause lower eGFR. Based on our MR study, these findings thus aid the notion that elevations of metabolites of the tryptophan pathway are a consequence rather than a cause of reduced eGFR. Further research is needed to specifically examine these relationships with respect to CKD progression among patients with existing CKD.

## Methods

**Figure 2:**
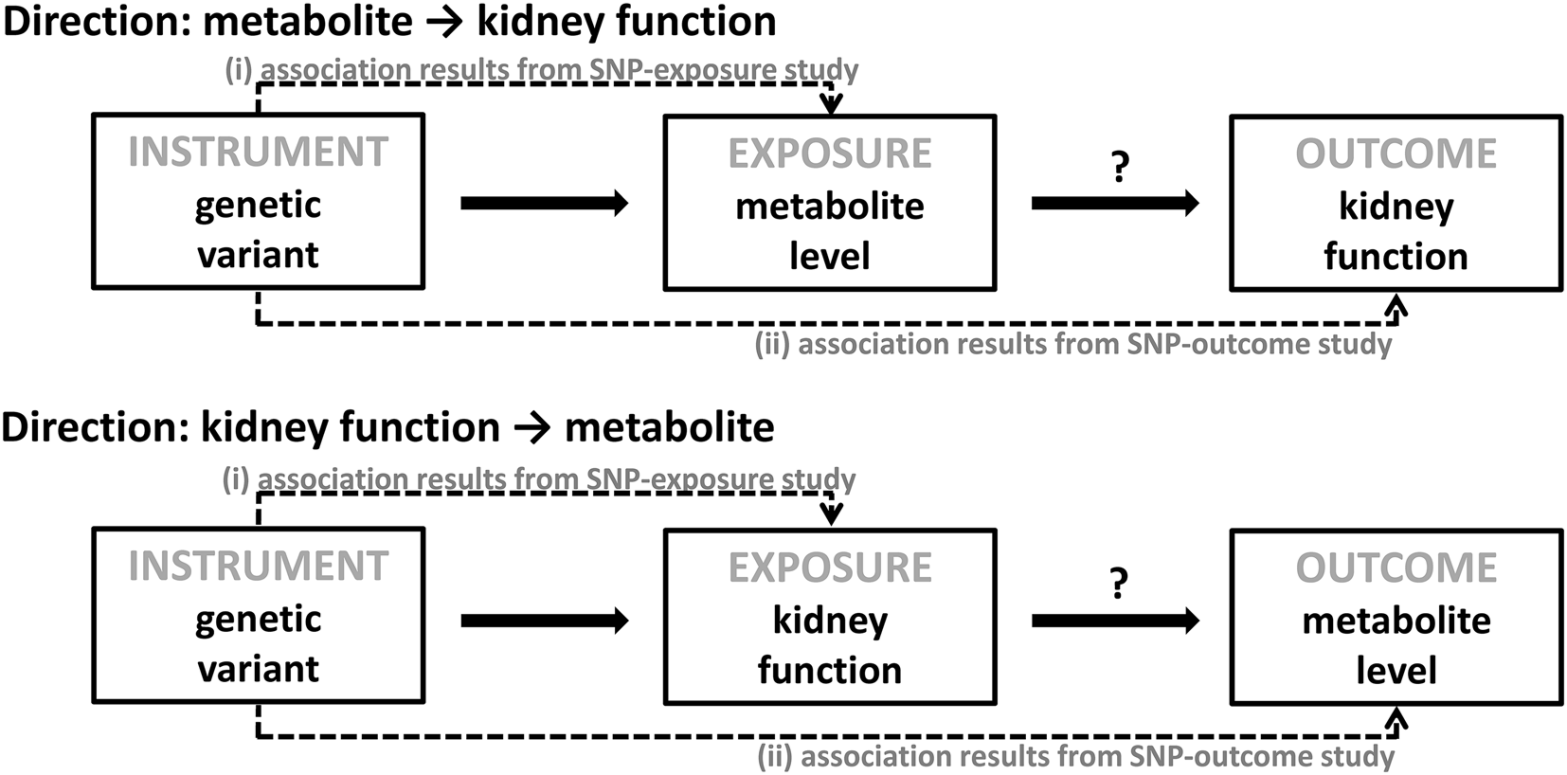
Analytical approach of Mendelian Randomization applied in this study.

A bidirectional MR approach was used to infer both directions, whether metabolite levels in blood causally affect kidney function, or whether kidney function levels causally affect metabolite levels in blood using publicly available genetic association summary data (two-sample MR)^17,23,24^. The underlying idea of the MR method is to use genetic variants as instruments to assign participants of a study to exposure groups in order to evaluate the causal relationship of this exposure with an outcome^82^. In this MR study, exposures and outcomes of interest for the direction: metabolite → kidney function, are blood metabolite levels of the tryptophan pathway and the kidney function marker eGFR, respectively (Fig. 2). For the other direction: kidney function → metabolite, the assignment is reversed. Since germline genetic variants are randomly assigned during gamete formation and represent a non-modifiable, lifelong exposure, the assignment to exposure groups using such variants as instruments conceptually corresponds to the randomization of participants in a clinical trial. For example, persons can be exposed to genetically determined higher or lower blood tryptophan levels over the course of their life. In order to represent a valid instrument, a genetic variant needs to fulfil three assumptions: (1) it must be associated with the exposure, (2) it must not be associated with confounders of the exposure-outcome relationship, and (3) it must only be associated with the outcome through the exposure^17^.

### Tryptophan pathway

Because of the research question addressing not just one metabolite but a whole pathway, a biology-centered literature search to identify metabolites of the tryptophan pathway in humans was conducted. Briefly, the basic tryptophan metabolism structure was obtained from the KEGG database^83^, and then each reaction was cross-checked in the Reactome database (tryptophan catabolism, URL with https://doi.org/10.3180/REACT_916.2; serotonin and melatonin biosynthesis, URL with StableID: R-HSA-209931; Nicotinate metabolism, URL with StableID: R-HSA-196807)^10,84,85^. While some typical metabolites related with tryptophan lack of direct reactions, we assembled all obtained information into Fig. 1. As a result, over 40 metabolites that qualified as downstream products of tryptophan were identified. A summarizing description of the pathway can be found in Supplementary Information S1.

### Selected studies

MR analyses were based on publicly available research results that reported on the association between genome-wide genetic variants with either metabolites of interest or eGFR, a measure of kidney function.

Since we were interested in several metabolites (Fig. 1), we evaluated genetic screens of many metabolites. Based on the results of a systematic literature search in December of 2017^5^, we identified studies that met the following pre-defined criteria: (1) evaluation of serum or plasma samples, (2) total population size of > 1,500 participants, (3) evaluation of at least one metabolite of interest, and (4) publicly available genome-wide association summary statistics. After exclusion of studies that were part of a more comprehensive study, four studies were selected (Supplementary Table 6)^86–89^. All four studies reported association results for a varying number of metabolites of interest (range: 1–13). Some metabolites were available in more than one study.

Regarding eGFR as a measure of kidney function, we used the most comprehensive results from a GWAS meta-analysis of eGFR published by the Chronic Kidney Disease Genetics (CKDGen) consortium in 2016 that were available at the time our MR study started^90^.

Basic characteristics of all five included studies are summarized in Supplementary Table 2. The number of single study groups contributing to the selected summary statistics varied greatly (range: 1–48). Some of the single study groups contributed to both, GWAS of metabolites and of eGFR. For example, data from the KORA F4 study were included into the GWAS of metabolites of Shin et al. (~ 23% of total study population) as well as into the GWAS of eGFR where its proportion to the whole study population is quite negligible (< 3%)^87,90^.

### Selection of instruments

Several steps were required to prepare data for the different analyses (Supplementary Information 2). Briefly, genetic instruments were SNPs with an association *p*-value < 5 × 10^–8^ (i.e. genome-wide significant) in the GWAS of the respective exposure that were also present in the GWAS of the respective outcome. In case several SNPs were available for an exposure, only independent SNPs were considered (linkage disequilibrium *R*^2^ < 0.2). After alignment and harmonization of data from different sources, data for ten metabolites (exposures) from three studies were available for the analysis addressing the direction: metabolite → kidney function^86,87,89^. For the other direction: kidney function → metabolite, only the study by Shin et al. contributed data of eight metabolites (outcomes)^87^, because the studies by Rhee et al*.* and Long et al. did not publish full genome-wide association summary statistics (Supplementary Table 2)^86,89^. Regarding eGFR, data for the analysis of both directions were available^90^.

### Statistical analysis

All analyses were conducted using R: A Language and Environment for Statistical Computing (https://www.R-project.org) in combination with the R package TwoSampleMR (MR-Base)^81^.

MR analysis was conducted for all exposures with at least one available instrument (Supplementary Table 3). In the main analysis, the effect for each instrument was estimated with the Wald ratio estimator and combined with the inverse variance weighted (IVW) meta-analysis method when several instruments were available^80^. To correct for multiple testing, statistical significance was defined using a Bonferroni correction that acknowledges the conduct of 18 MR analyses (metabolite → kidney function: 10; kidney function → metabolite: 8): 0.05/(10 + 8) = 2.78E−03. While in the main analysis, all selected instruments were used, the analyses were repeated in a sensitivity analysis with instruments that were selected based on a more stringent pruning (LD *R*^2^ < 0.001).

For all MR analyses utilizing more than three instruments, sensitivity analyses were conducted in order to evaluate the robustness of results and to assess different potential violations of the assumptions underlying MR, using additional estimation methods as provided by the TwoSampleMR-package such as median-based estimation and Egger regression^81^. In addition, the MR-PRESSO method was applied to detect outliers and to provide an outlier-corrected effect estimate^91^. An overview of all estimation methods is provided in Supplementary Information 3.

Furthermore, we annotated all selected genetic instruments for their associations with other complex traits and diseases using the comprehensive SNiPA database as well as GeneATLAS^92,93^. This annotation allowed for the evaluation of potential pleiotropy from a non-statistical perspective. Based on the obtained annotations, we defined two subsets of SNPs to minimize the risk of bias due to pleiotropy (Supplementary Table 5, Selection 1 [less stringent] and 2 [more stringent]). In addition, we used subsets of SNPs with very strong associations with the respective trait (*p* < 10^–20^ and *p* < 10^–10^) to evaluate potential bias due to the inclusion of weaker instruments (Supplementary Table 5, Selection 3). For eGFR as exposure, we specifically used another subset of eGFR instruments that was restricted to replicated SNPs to minimize the chance of including SNPs with false-positive associations (Supplementary Table 5, Selection 4). Respective MR analyses were conducted using the Wald ratio estimator (1 instrument) or the IVW estimator (> 1 instrument).

Finally, we excluded single variants selected for several traits or in LD (*R*^2^ > 0.2) with other variants selected for other traits in a separate sensitivity analysis whenever possible.

## Supplementary information

Supplementary file 1

Supplementary file 2
