# Supplementary material for: The relationship between blood metabolites of the tryptophan pathway and kidney function: a bidirectional Mendelian randomization analysis
Source: Sci Rep. 2020 Jul 29;10:12675. doi: 10.1038/s41598-020-69559-x (PMC7391729; doi:10.1038/s41598-020-69559-x)
Supplement: Supplementary file 1 [file 41598_2020_69559_MOESM1_ESM.docx]

**The Relationship between Blood Metabolites of the Tryptophan Pathway and Kidney Function: A Bidirectional Mendelian Randomization Analysis**

Yurong Cheng, Yong Li, Paula Benkowitz, Claudia Lamina, Anna Köttgen, Peggy Sekula

**Supplementary Material**

**Contents**

**Supplementary Information**

**Supplementary Information 1:** Tryptophan pathway – An overview

**Supplementary Information 2:** Preparation of retrieved summary statistics for analysis

**Supplementary Information 3:** Statistical methods to estimate and to validate causal effects

**References**

**Supplementary Figures**

**Supplementary Figure 1:** Reported correlations between evaluated metabolites

**Supplementary Figure 2:** Forest plots for leave-one-out analyses

**Supplementary Figure 3:** Results of different analysis approaches for the four significant associations observed in main analysis

**Supplementary Tables (*see extra xlsx-file*)**

**Supplementary Table 1:** Selected studies reporting on associations of metabolites of tryptophan pathway with kidney function or disease

**Supplementary Table 2:** Description of the selected studies

**Supplementary Table 3:** Overview of SNPs selected as genetic instruments in various analyses

**Supplementary Table 4:** Results of conducted main and sensitivity analyses

**Supplementary Table 5:** Detailed information on all genetic instruments used in any MR analysis and definition of subsets

**Supplementary Table 6:** Identified genome-wide association studies of metabolic traits

**Supplementary Information 1: Tryptophan pathway – An overview**

Tryptophan is an essential aromatic amino acid containing an indole group; it is the least abundant of the amino acids, with the largest molecular weight^1^. Its intestinal absorption and efflux are largely mediated through B^0^AT1 (SLC6A19) and SLC16A10^2-5^. While less than 1% of tryptophan is actually used for protein biosynthesis, the major proportion serves as a biosynthetic precursor for microbial and host metabolites of different pathways (see **Figure 1** in main manuscript)^1,6^:

Most of the ingested tryptophan (~95%) feeds into the kynurenine pathway^7^. The two enzymes indoleamine 2,3-dioxygenase (IDO), widely distributed in extrahepatic tissues, and tryptophan 2,3-dioxygenase (TDO), mainly present in the liver, convert tryptophan to N-formyl-kynurenine in a first rate-limiting step^7-9^.

Another part of the tryptophan (1-2%) is used to produce the neurotransmitter serotonin^10^. In the brain, serotonin is produced through the tryptophan hydroxylase 2 enzyme (TpH2); in the body, most serotonin is produced in the gut and particularly in enterochromaffin cells through the tryptophan hydroxylase 1 enzyme (TpH1). Both, TpH1 and TpH2, are rate-limiting enzymes for serotonin synthesis^9,11,12^. As peripheral 5-hydroxytryptophan does not cross the blood-brain barrier, it triggers numerous functions in the gastrointestinal tract through activating specific 5-hydroxyryptamine (5-HT) receptors^12^.

Another part of the ingested tryptophan (4-6%) is degraded by gut microbiota, and then catalysed through the gut or liver path^7,10^. Recent discoveries underscored the importance of this pathway, especially as gut microbiota may limit tryptophan availability for the host^13^. Although the measurements of the derivatives related with indole and identification of intestinal microbiome are not difficult at the technical level, it is still complicated to determine which paths of indole metabolites are involved. As one tryptophan metabolite can be produced by mutual cooperation of different bacteria offering catalytic enzymes, incomplete knowledge about the coordination of different species of bacteria in the generation of tryptophan metabolites as well as about differences between individuals in gut microbiota and intestinal environment brings challenges to illuminate tryptophan metabolism^13^. In addition, some tryptophan is modulated into indole derived through endogenous enzymes for endocrine regulation^7,14^.

Lastly, a minor proportion of tryptophan (~0.5%) is excreted unchanged into urine^10^.

**Supplementary Information 2: Preparation of retrieved summary statistics for analysis**

Several steps were required to prepare data of the selected studies^15-19^:





First of all, genetic association summary statistics were lifted to hg19 (build37) using the web interface LiftOver (https://genome.ucsc.edu/ cgi-bin/hgLiftOver) if necessary. Subsequently, data to address the two different parts of the research question were prepared separately. While the exposures of interest were the metabolites and the outcome was kidney function for one direction, the exposure of interest was kidney function and the outcomes were the metabolites for the other. Genetic instruments for the respective exposure were selected from the relevant genome-wide association studies.

Per exposure and data source, potential instruments were all SNPs with an association *p*-value<5×10^-8^ (i.e. genome-wide significant). In order to maximize the number of potential instrumental variables, we then assessed which of the SNPs including proxies (linkage disequilibrium [LD] *R²*>0.8) were available in the respective outcome GWAS. Only afterwards, the set of potential instruments was pruned to a set of independent markers using Plink v1.90 (options: r^2^ 0.2, window: 1000 kb, significance threshold for index SNP 5×10^−8^)^20^. Post-hoc check of LD of selected SNPs per trait led to the exclusion of one SNP for kynurenine because pairwise LD was >0.2. The SNP with the lower *p*-value was kept. Finally, selected association results were harmonized with respect to the coded allele using the 1000 Genomes project phase 3 EUR as the reference.

Despite the comprehensive evaluation of SNPs, for some reported exposures no instrument could be identified. For the evaluation of the impact of metabolites on eGFR, data for ten metabolites (exposures) from three studies, excluding Draisma *et al*., were available to be related to the kidney function measure eGFR^15-18^. Only the study by Shin *et al*. contributed data of eight metabolites (outcomes) for the evaluation of the other direction^16^, because the studies by Rhee *et al.* and Long *et al*. did not publish full genome-wide association results that could be used as outcome (**Supplementary Table 2**)^15,18^.

As sensitivity analysis, the above described pruning was repeated with a more stringent threshold of LD *R²*<0.001.

**Supplementary Information 3: Statistical methods to estimate and to validate causal effects**

| **Estimation methods** | **Comments** | **Reference** |
| --- | --- | --- |
| **# instruments = 1** | | |
| Wald ratio test | - standard method to estimate causal effect for a single instrument using summarized data: ${\hat{\beta}_{outcome\vert IV}}/{\hat{\beta}_{exposure\vert IV}}$ | ^21,22^ |
| **# instruments > 1** | | |
| Inverse variance weighted (IVW) | - combines the variant-specific ratio estimates using a fixed‐effect meta‐analysis - most efficient, greatest power - assumption: all variants are valid IVs, balanced pleiotropy - biased, if average pleiotropic effect differs from zero - often used in the main analysis | ^23,24^ |
| Egger | - requires at least three genetic variants - allows all genetic variants to have pleiotropic effects - assumption: pleiotropic effects are independent of variant-exposure associations (InSIDE assumption, often not plausible) - sensitive to influential datapoints and violations of InSIDE assumption | ^23,25^ |
| Simple/weighted mode | - maximum value of the kernel-weighted density of all single variant-specific causal estimates - assumption: # variants estimating true causal effect >   # variants estimating any other effect - robust against outlying variant-specific estimates/ pleiotropic variants, if they all estimate different causal effects - versions: unweighted or weighted (inverse variance) | ^23,26^ |
| Weighted median | - calculates median from variant-specific casual estimates (ratio method) - weights: inverse variance - assumption: >50% of instruments are valid instruments - robust against small number of outlying variant-specific estimates/pleiotropic variants | ^23,27^ |
| Leave-one-out | - assesses reliance on a single variant - iterative estimation of casual effects based on all IVs except one using Wald ratio test or IVW - IVW is used to obtain overall estimate from all leave-one-out estimates | ^28^ |
| Egger intercept | - estimate for the average pleiotropic effect - Egger intercept=0 🡪 MR-Egger = IVW estimate - Egger intercept≠0 🡪 IVW estimate likely biased | ^25^ |
| Heterogeneity Q | - assesses heterogeneity between variant-specific ratio estimates - small *p*-value indicates heterogeneity between IVs | ^29^ |
| MR-PRESSO global test | - requires more than three genetic variants - test to detect horizontal pleiotropy - similar to heterogeneity estimate | ^30^ |
| MR-PRESSO estimate | - removes single variants with outlying variant-specific causal estimates (based on heterogeneity measures) und then uses IVW - efficient for valid IVs - high false-positive rate when several invalid IVs are present | ^23,30^ |

IV: Instrumental Variable, IVW: inverse variance weighted

**References**

1 Alkhalaf, L. M. & Ryan, K. S. Biosynthetic manipulation of tryptophan in bacteria: pathways and mechanisms. *Chem Biol* **22**, 317-328, doi:10.1016/j.chembiol.2015.02.005 (2015).

2 Broer, A. *et al.* Molecular cloning of mouse amino acid transport system B0, a neutral amino acid transporter related to Hartnup disorder. *J Biol Chem* **279**, 24467-24476, doi:10.1074/jbc.M400904200 (2004).

3 Kleta, R. *et al.* Mutations in SLC6A19, encoding B0AT1, cause Hartnup disorder. *Nat Genet* **36**, 999-1002, doi:10.1038/ng1405 (2004).

4 Kim, D. K. *et al.* Expression cloning of a Na+-independent aromatic amino acid transporter with structural similarity to H+/monocarboxylate transporters. *J Biol Chem* **276**, 17221-17228, doi:10.1074/jbc.M009462200 (2001).

5 Ramadan, T. *et al.* Basolateral aromatic amino acid transporter TAT1 (Slc16a10) functions as an efflux pathway. *J Cell Physiol* **206**, 771-779, doi:10.1002/jcp.20531 (2006).

6 Strasser, B., Gostner, J. M. & Fuchs, D. Mood, food, and cognition: role of tryptophan and serotonin. *Curr Opin Clin Nutr Metab Care* **19**, 55-61, doi:10.1097/MCO.0000000000000237 (2016).

7 Peters, J. C. Tryptophan nutrition and metabolism: an overview. *Adv Exp Med Biol* **294**, 345-358, doi:10.1007/978-1-4684-5952-4_32 (1991).

8 Saito, K. *et al.* Mechanism of increases in L-kynurenine and quinolinic acid in renal insufficiency. *Am J Physiol Renal Physiol* **279**, F565-572, doi:10.1152/ajprenal.2000.279.3.F565 (2000).

9 Keszthelyi, D., Troost, F. J. & Masclee, A. A. Understanding the role of tryptophan and serotonin metabolism in gastrointestinal function. *Neurogastroenterol Motil* **21**, 1239-1249, doi:10.1111/j.1365-2982.2009.01370.x (2009).

10 Bender, D. A. Biochemistry of tryptophan in health and disease. *Mol Aspects Med* **6**, 101-197 (1983).

11 Walther, D. J. *et al.* Synthesis of serotonin by a second tryptophan hydroxylase isoform. *Science* **299**, 76, doi:10.1126/science.1078197 (2003).

12 Mawe, G. M. & Hoffman, J. M. Serotonin signalling in the gut--functions, dysfunctions and therapeutic targets. *Nat Rev Gastroenterol Hepatol* **10**, 473-486, doi:10.1038/nrgastro.2013.105 (2013).

13 Gao, J. *et al.* Impact of the gut microbiota on intestinal immunity mediated by tryptophan metabolism. *Front Cell Infect Microbiol* **8**, 13, doi:10.3389/fcimb.2018.00013 (2018).

14 Russo, S. *et al.* Tryptophan as a link between psychopathology and somatic states. *Psychosom Med* **65**, 665-671, doi:10.1097/01.Psy.0000078188.74020.Cc (2003).

15 Rhee, E. P. *et al.* A genome-wide association study of the human metabolome in a community-based cohort. *Cell Metab* **18**, 130-143, doi:10.1016/j.cmet.2013.06.013 (2013).

16 Shin, S. Y. *et al.* An atlas of genetic influences on human blood metabolites. *Nat Genet* **46**, 543-550, doi:10.1038/ng.2982 (2014).

17 Draisma, H. H. M. *et al.* Genome-wide association study identifies novel genetic variants contributing to variation in blood metabolite levels. *Nat Commun* **6**, 7208, doi:10.1038/ncomms8208 (2015).

18 Long, T. *et al.* Whole-genome sequencing identifies common-to-rare variants associated with human blood metabolites. *Nat Genet* **49**, 568-578, doi:10.1038/ng.3809 (2017).

19 Pattaro, C. *et al.* Genetic associations at 53 loci highlight cell types and biological pathways relevant for kidney function. *Nat Commun* **7**, doi:10.1038/ncomms10023 (2016).

20 Chang, C. C. *et al.* Second-generation PLINK: rising to the challenge of larger and richer datasets. *Gigascience* **4**, doi:10.1186/s13742-015-0047-8 (2015).

21 Wald, A. The fitting of straight lines if both variables are subject to error. *Ann. Math. Statist.* **11**, 284-300, doi:10.1214/aoms/1177731868 (1940).

22 Burgess, S., Small, D. S. & Thompson, S. G. A review of instrumental variable estimators for Mendelian randomization. *Stat Methods Med Res* **26**, 2333-2355, doi:10.1177/0962280215597579 (2017).

23 Burgess, S. *et al.* Guidelines for performing Mendelian randomization investigations. *Wellcome Open Res* **4**, 186 (2019).

24 Burgess, S., Butterworth, A. & Thompson, S. G. Mendelian randomization analysis with multiple genetic variants using summarized data. *Genet Epidemiol* **37**, 658-665, doi:10.1002/gepi.21758 (2013).

25 Bowden, J., Smith, G. D. & Burgess, S. Mendelian randomization with invalid instruments: effect estimation and bias detection through Egger regression. *Int J Epidemiol* **44**, 512-525, doi:10.1093/ije/dyv080 (2015).

26 Hartwig, F. P., Davey Smith, G. & Bowden, J. Robust inference in summary data Mendelian randomization via the zero modal pleiotropy assumption. *Int J Epidemiol* **46**, 1985-1998, doi:10.1093/ije/dyx102 (2017).

27 Bowden, J., Smith, G. D., Haycock, P. C. & Burgess, S. Consistent Estimation in Mendelian Randomization with Some Invalid Instruments Using a Weighted Median Estimator. *Genet Epidemiol* **40**, 304-314, doi:10.1002/gepi.21965 (2016).

28 Burgess, S., Bowden, J., Fall, T., Ingelsson, E. & Thompson, S. G. Sensitivity Analyses for Robust Causal Inference from Mendelian Randomization Analyses with Multiple Genetic Variants. *Epidemiology* **28**, 30-42, doi:10.1097/ede.0000000000000559 (2017).

29 Bowden, J., Hemani, G. & Davey Smith, G. Invited Commentary: Detecting Individual and Global Horizontal Pleiotropy in Mendelian Randomization-A Job for the Humble Heterogeneity Statistic? *Am J Epidemiol* **187**, 2681-2685, doi:10.1093/aje/kwy185 (2018).

30 Verbanck, M., Chen, C. Y., Neale, B. & Do, R. Detection of widespread horizontal pleiotropy in causal relationships inferred from Mendelian randomization between complex traits and diseases. *Nat Genet* **50**, 693-698, doi:10.1038/s41588-018-0099-7 (2018).

**Supplementary Figure 1: Reported correlations between evaluated metabolites**

NA: not available Color legend: Correlation coefficient (Pearson/Spearman)

**Supplementary Figure 2: Forest plots for leave-one-out analyses**

**(A) kynurenine → eGFR (5 instruments) (B) tryptophan → eGFR (22 instruments)**

**
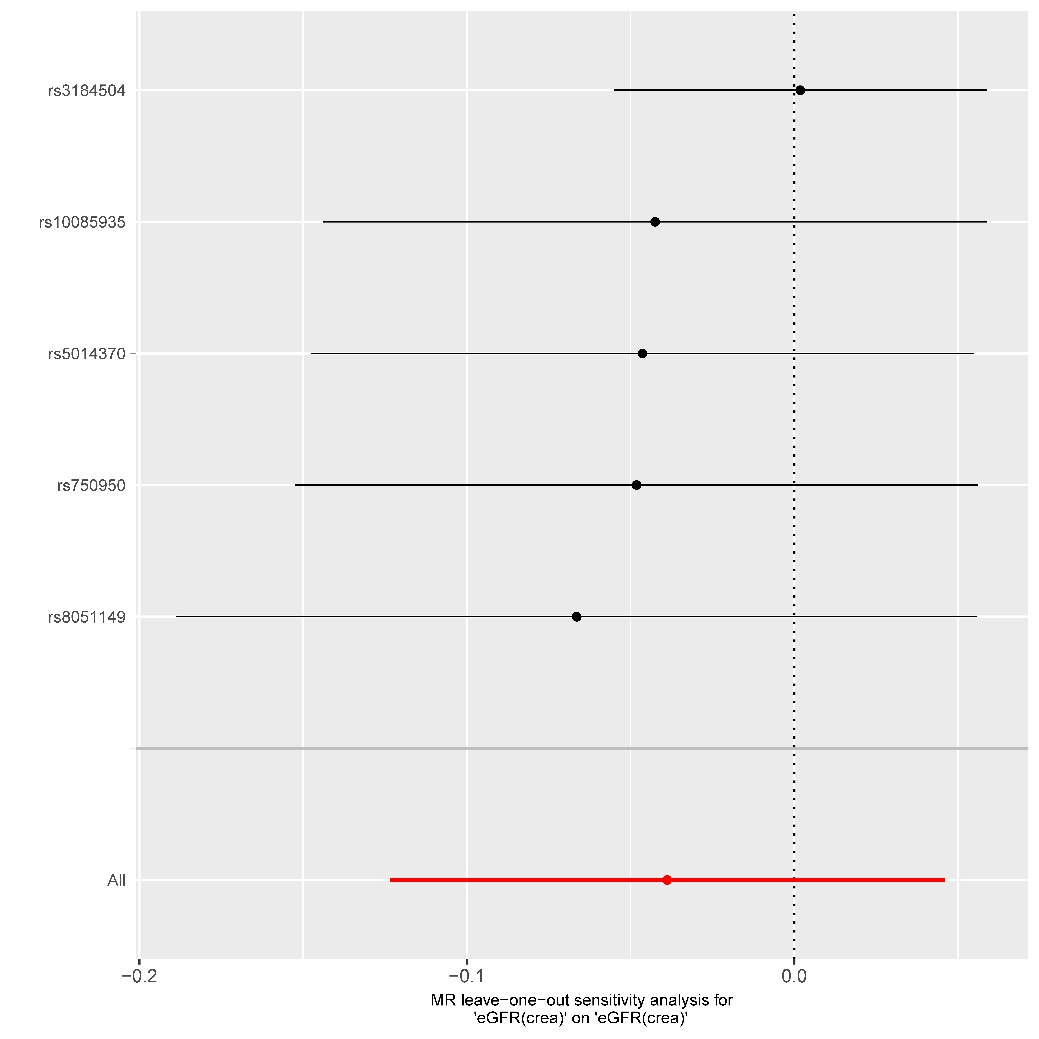

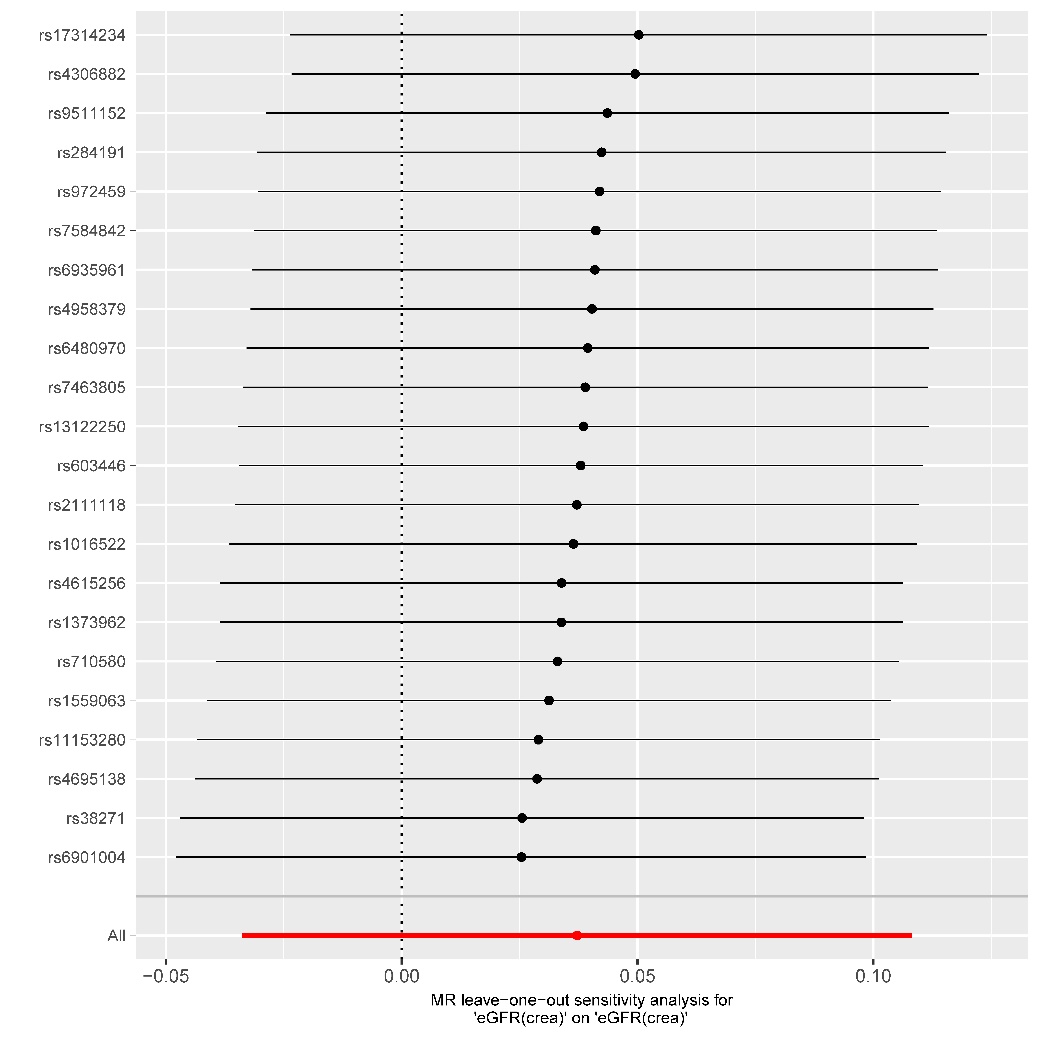
**

**(C) eGFR → 3-indoxyl sulfate (73 instruments) (D) eGFR → C-glycosyltryptophan (73 instruments)**

**
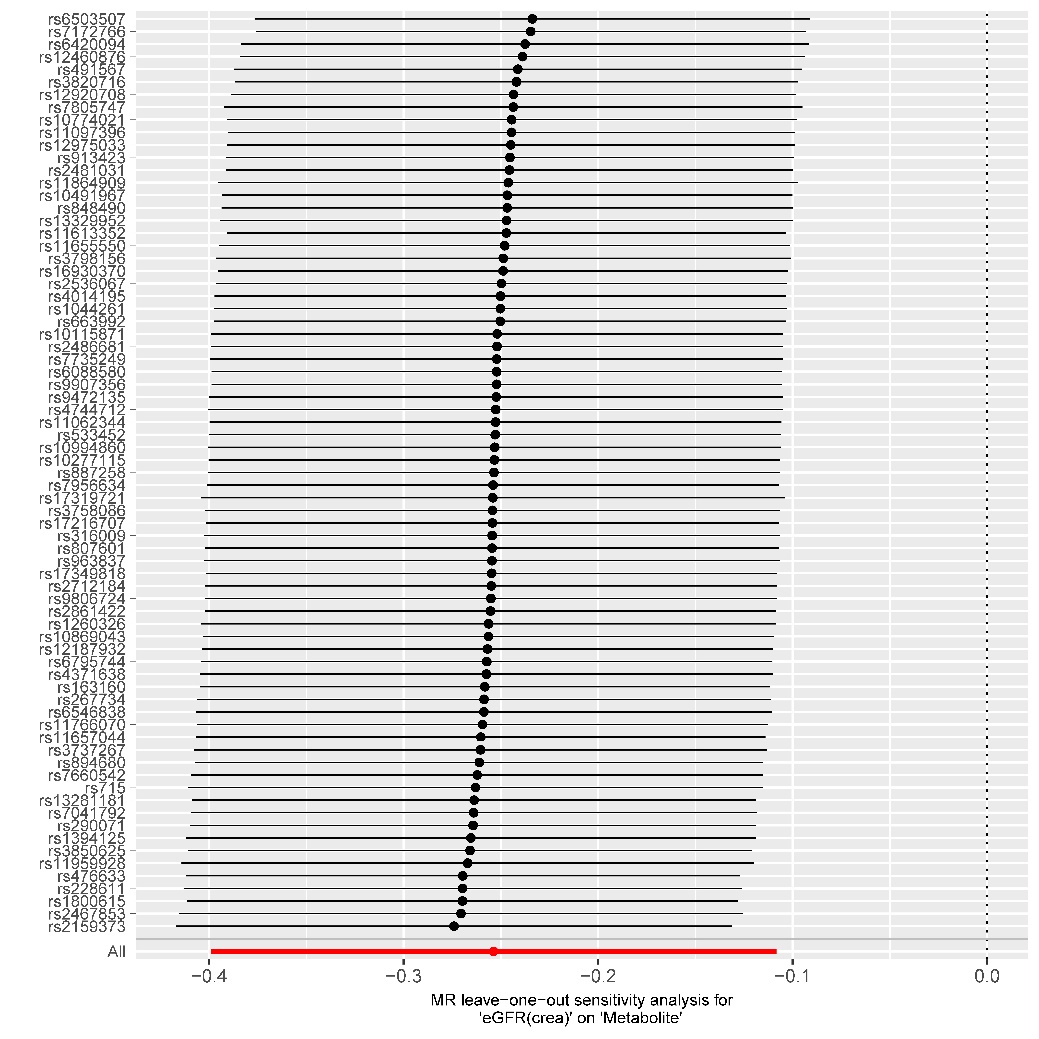

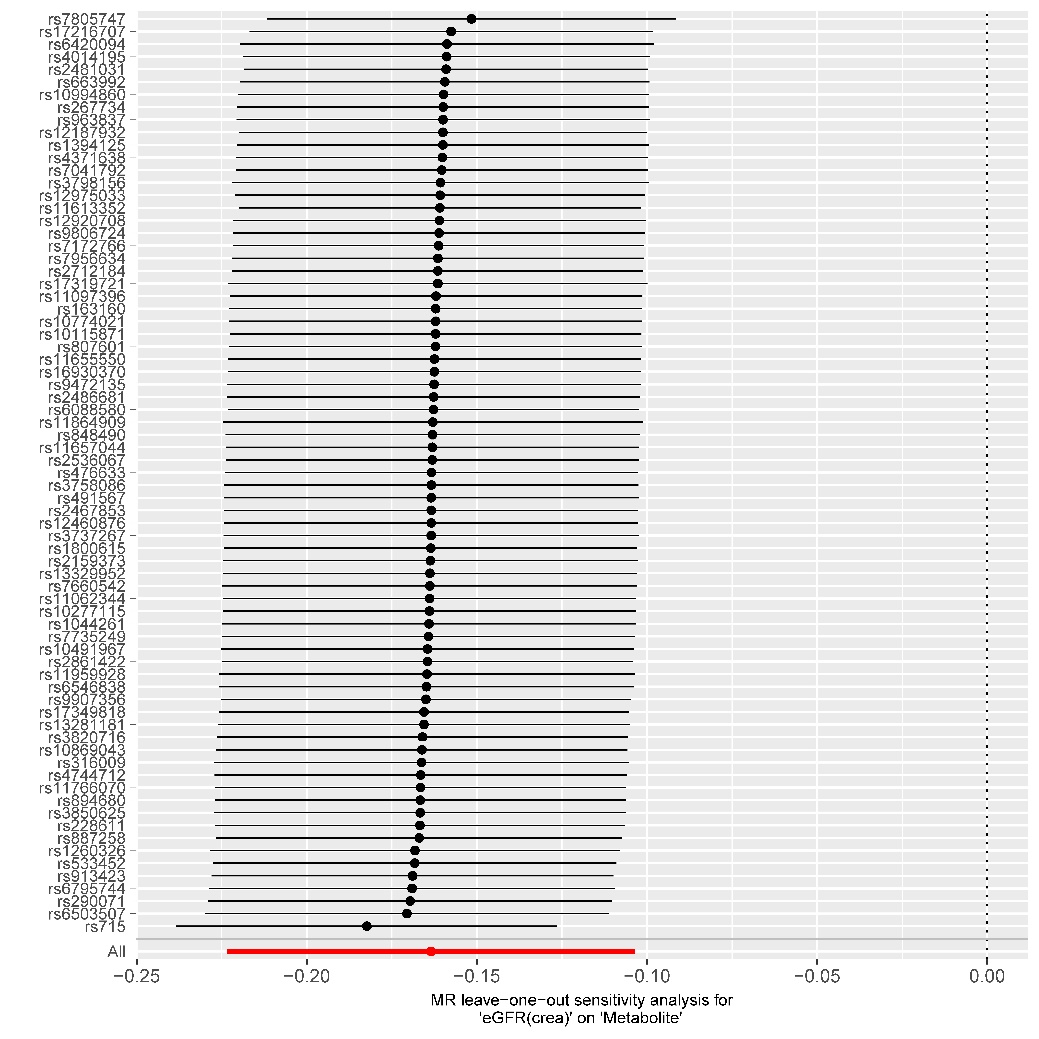
**

**(E) eGFR → indoleacetate (73 instruments) (F) eGFR → indole-3-lactate (73 instruments)**

**
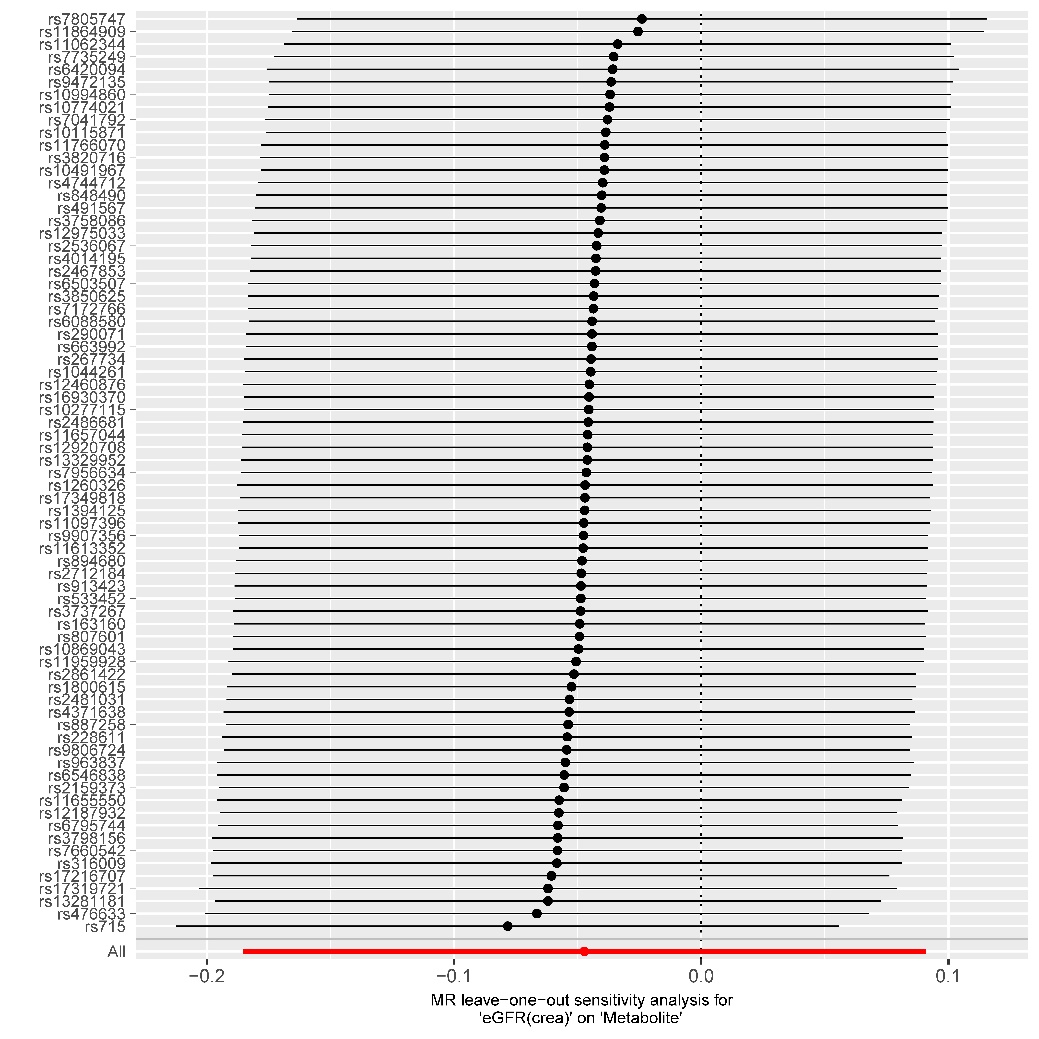

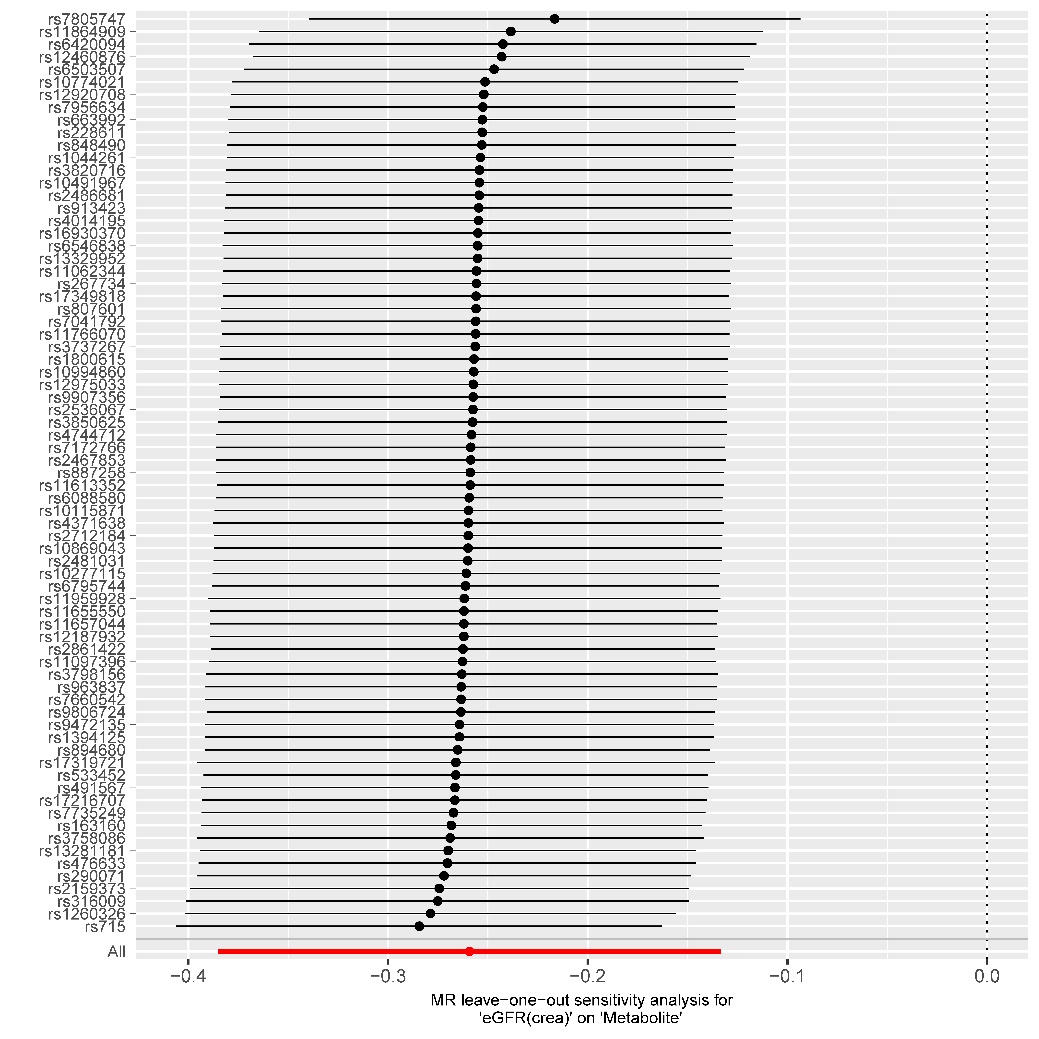
**

**(G) eGFR → indole-3-propionate (73 instruments) (H) eGFR → kynurenine (73 instruments)**

**
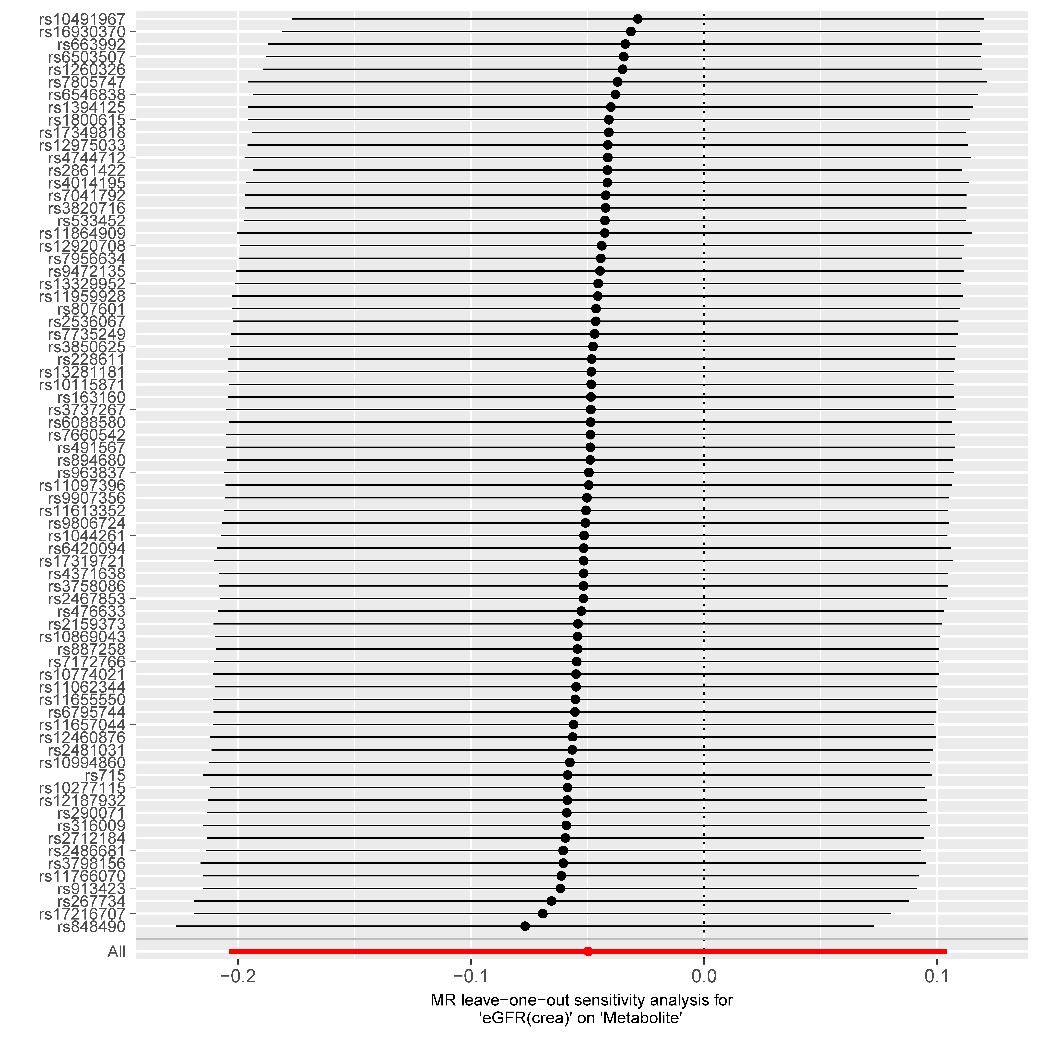

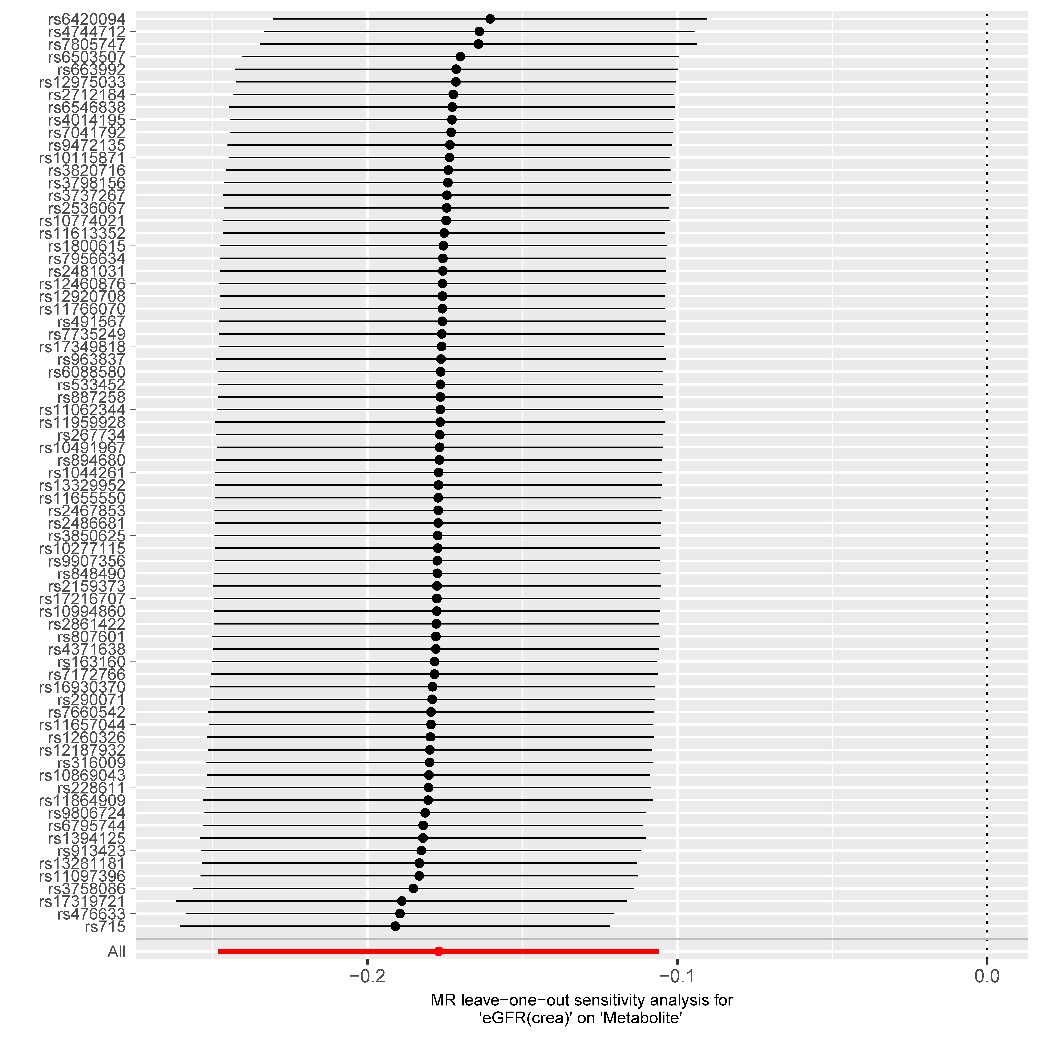
**

**(I) eGFR → serotonin (73 instruments) (J) eGFR → tryptophan (73 instruments)**

**
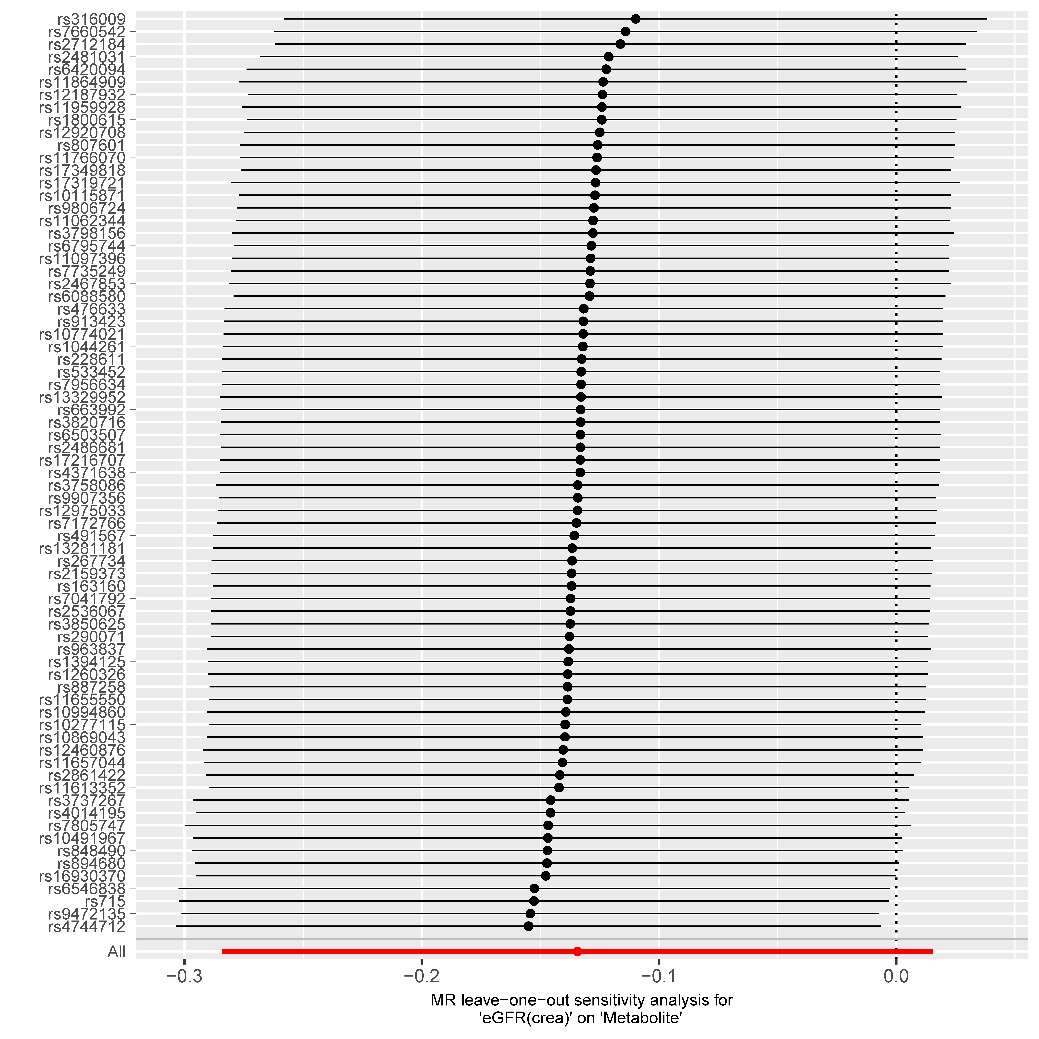

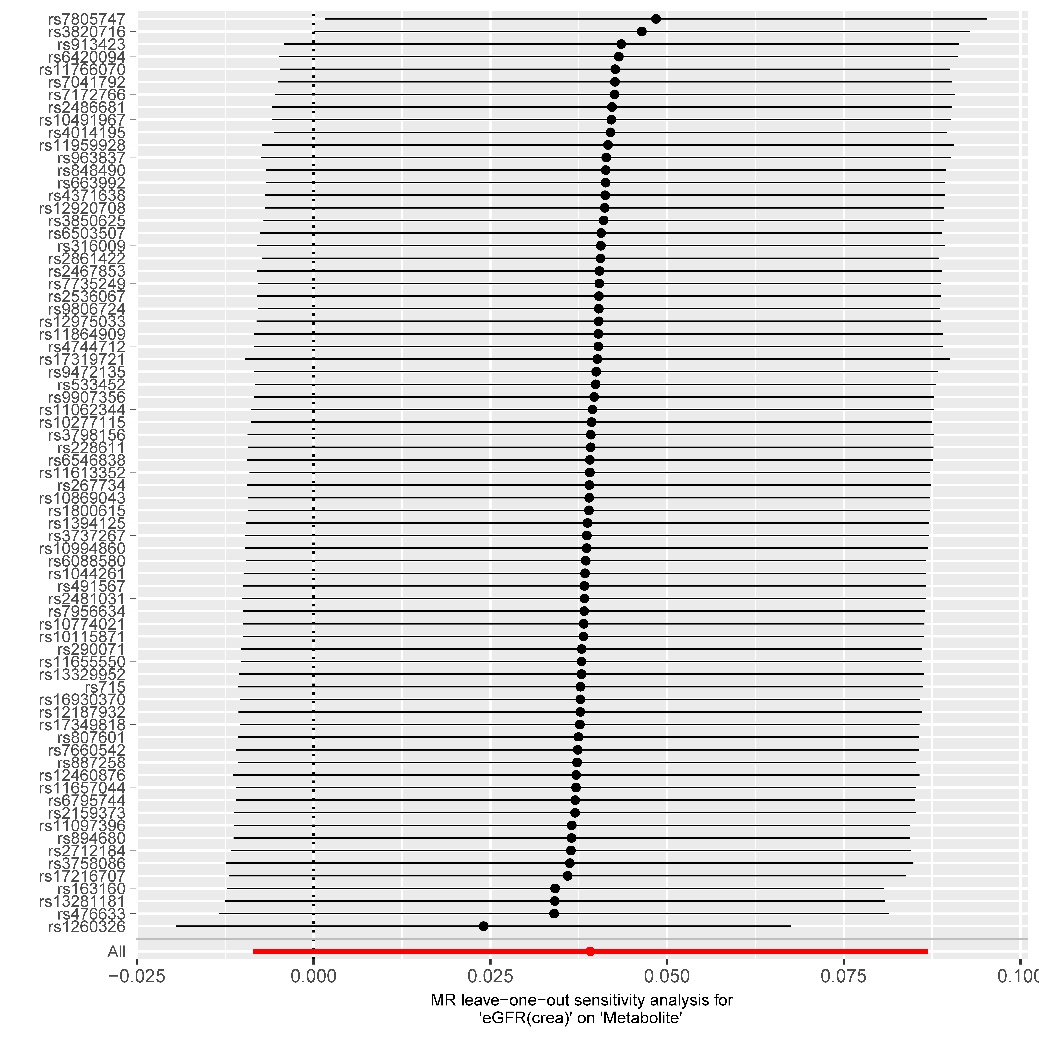
**

**Supplementary Figure 3: Results of different analysis approaches for the four significant associations observed in main analysis**

**(A) kynurenine (B) C-glycosyltryptophan**


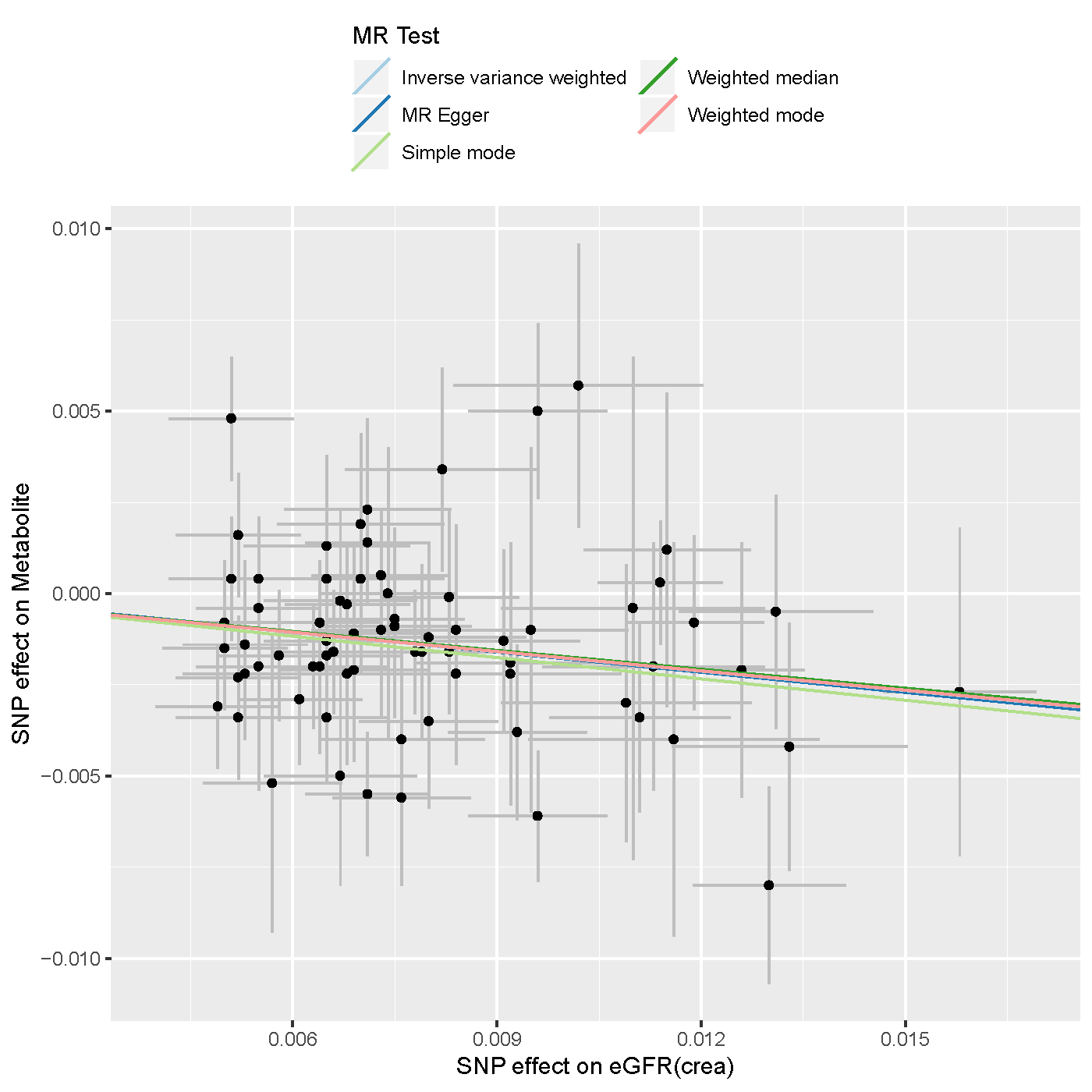

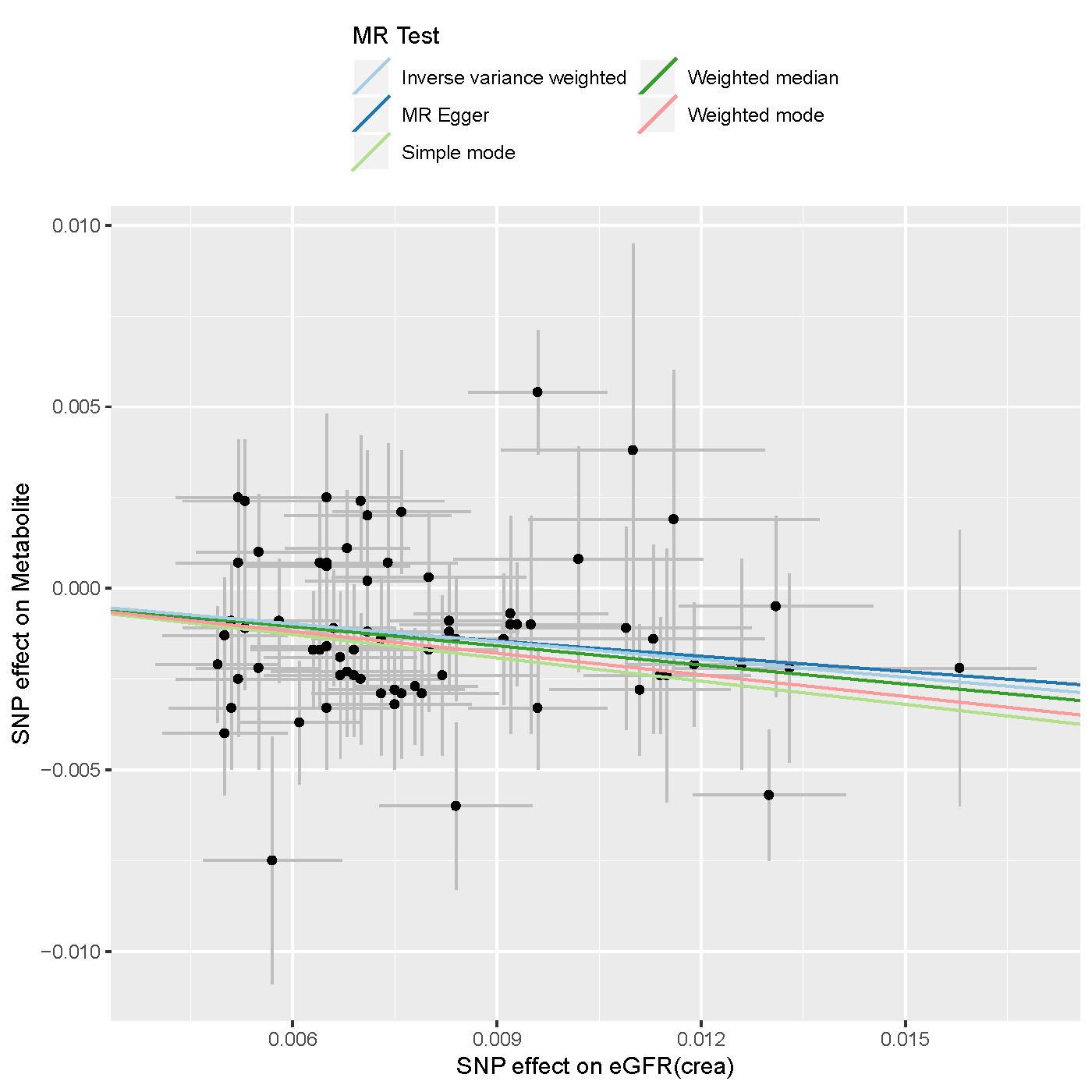


**(C) 3-indoxyl sulfate (D) indole-3-lactate**


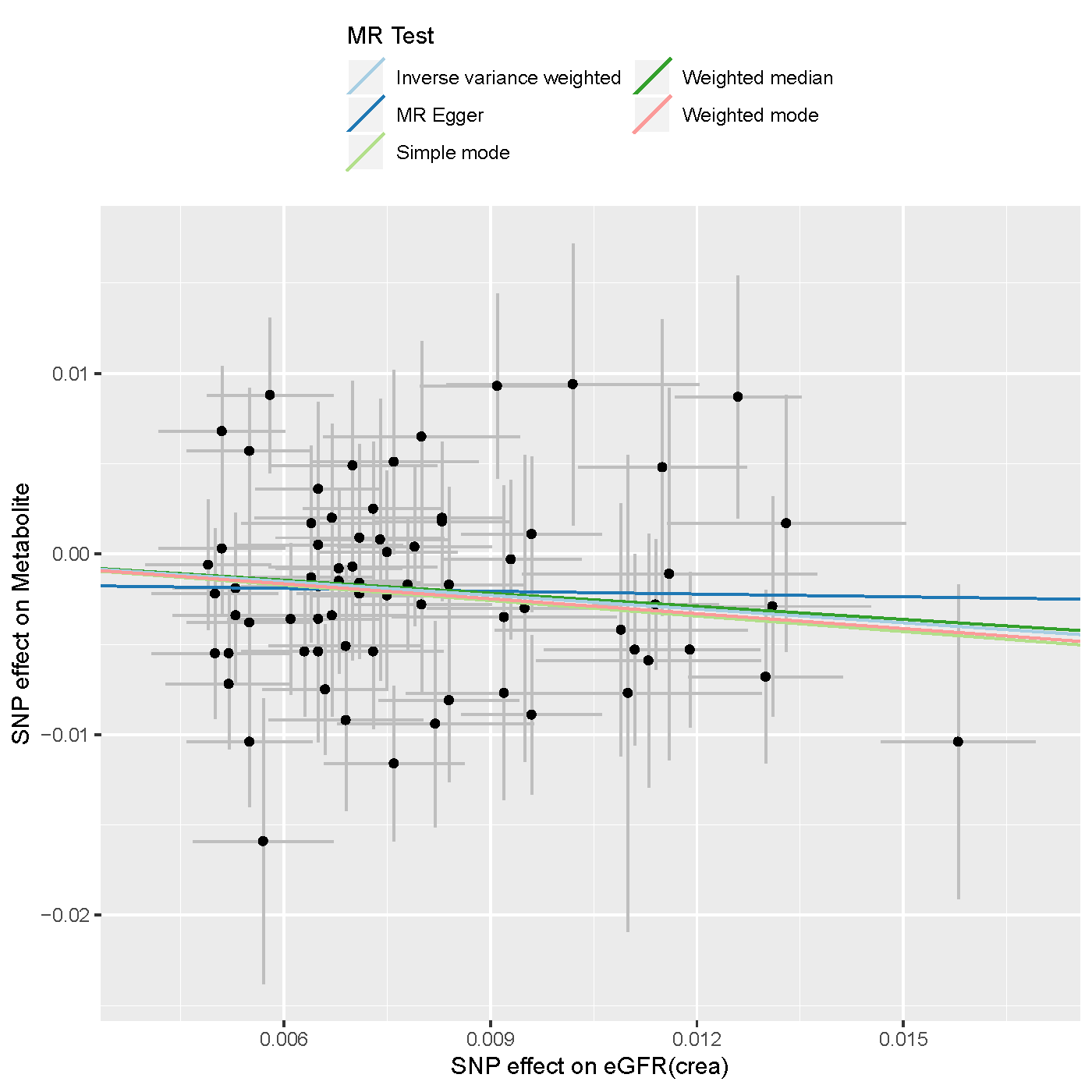

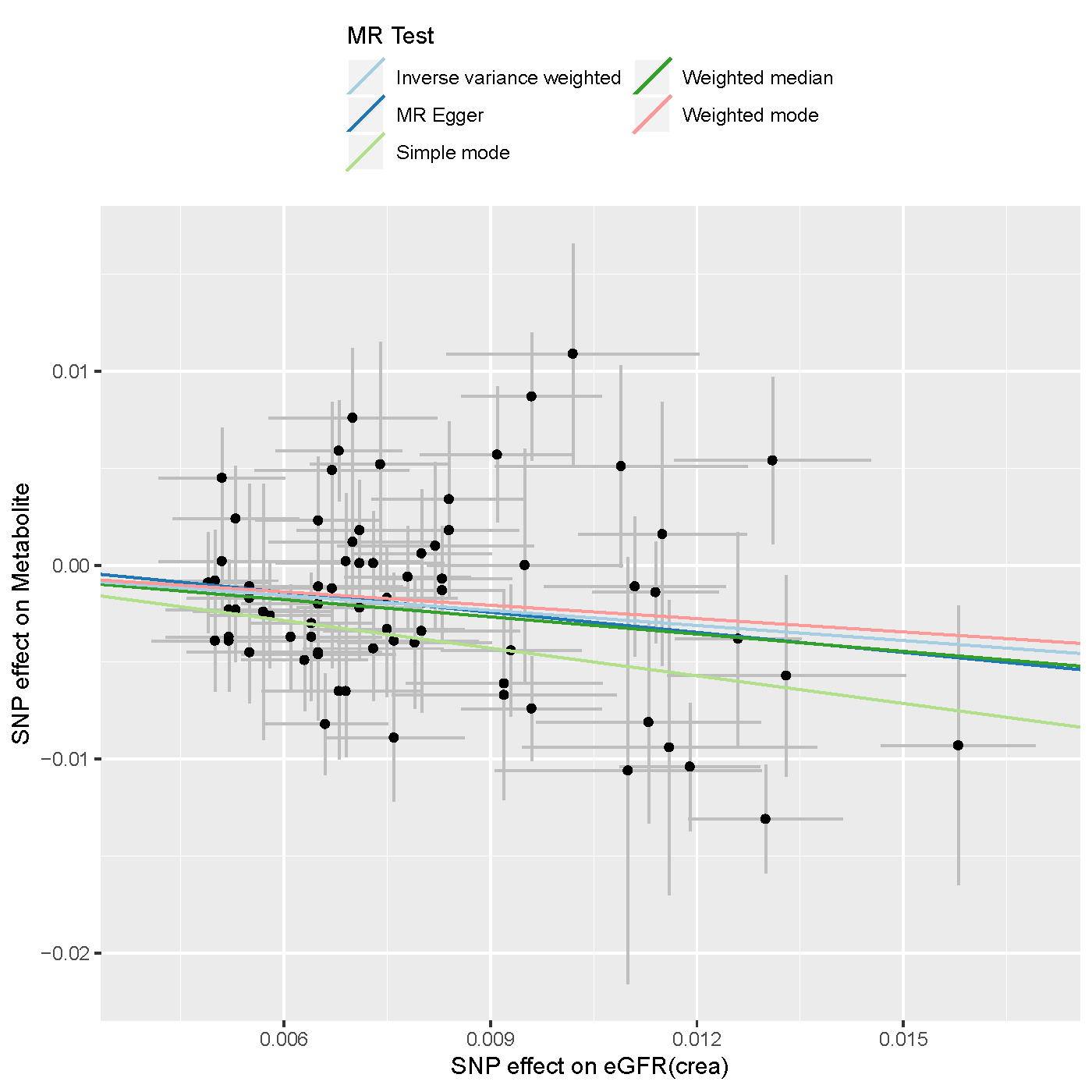


2

1

For the different metabolites with significant associations in the main analysis, the figures display reported effects and standard errors of the single instrumental SNPs with respect to their associations with eGFR (x-axis) and metabolite (y-axis). The slopes of the included lines correspond to estimates using various estimation methods (main analysis = inverse variance weighted method). For indole-3-lactate (D), the two red circles additionally indicate the two SNPs detected as outlier by MR-PRESSO (1: rs715, 2: rs7805747).
